# Periodontal inflammation as a potential driver of HIV low level viremia

**DOI:** 10.1371/journal.pone.0305641

**Published:** 2024-06-17

**Authors:** Arjen J. Stam, Hester Groenewegen, Arjan Vissink, Annemarie M. J. Wensing, Monique Nijhuis, Wouter F. W. Bierman

**Affiliations:** 1 Department of Medical Microbiology, Translational Virology, University Medical Center Utrecht, Utrecht, The Netherlands; 2 Department of Infectious Diseases, Public Health Service Amsterdam, Amsterdam, The Netherlands; 3 Department of Oral and Maxillofacial Surgery, University Medical Center Groningen and University of Groningen, Groningen, The Netherlands; 4 Department of Health, Ezintsha, University of the Witwatersrand, Johannesburg, South Africa; 5 Department of Internal Medicine, Division of Infectious Diseases, University Medical Center Groningen and University of Groningen, Groningen, The Netherlands; Nigerian Institute of Medical Research, NIGERIA

## Abstract

HIV can be successfully suppressed to undetectable levels by antiretroviral therapy (ART) in most people with HIV (PWH). However, a small proportion continues to have persistent low-level viremia (LLV) during ART. A presumed source of LLV is production or replication from viral reservoirs, which are maintained in the presence of ART. It is unknown whether the oral cavity can be considered an HIV reservoir. As periodontal inflammation is a common problem in PWH, we hypothesize that periodontal inflammation in the oral cavity activates (latently) infected cells and thus might be associated with LLV. We included 11 individuals with HIV LLV, and compared HIV-RNA levels in saliva and plasma at baseline and at week 24 after switch of ART. We compared the LLV-group at baseline with 11 age-matched controls with suppressed viremia. To investigate the severity of periodontitis we used Periodontal Inflamed Surface Areas (PISA) by measuring probing depth, gingival recession, bleeding on probing and clinical attachment level. Severity of periodontitis was classified according to the CDC-AAP case definition. Additional insights in periodontal inflammation were obtained by comparing immune activation markers and the presence of periodontal pathogens. In four individuals of the LLV group, residual levels of HIV-RNA were detected in saliva at baseline (N = 1) or at week 24 (N = 2) or both (N = 1). Of the four individuals with LLV, three had residual levels of HIV-RNA in saliva. All 22 individuals had moderate to severe periodontitis. PISA was not significantly different between cases with LLV and controls. Similarly, periodontal pathogens were frequently observed in both groups. Total activated HLA-DR^+^CD38^*+*^ CD4^+^ cells and CD8^+^ cells were significantly higher in the LLV group than in the control group (p = <0.01). No immune markers were associated with LLV. In conclusion, periodontal inflammation is an unlikely driver of HIV LLV compared to HIV suppressed individuals.

## Introduction

Periodontitis is a chronic inflammatory disease characterized by the destruction of the supporting structures of the teeth, including the gums, periodontal ligament, and alveolar bone, as a result of bacterial infection [1] and can range from mild gingivitis to the more severe periodontitis. People with HIV (PWH) on antiretroviral treatment (ART) are known to have a higher prevalence of periodontitis compared to the general population [2] Other oral diseases such as necrotizing ulcerative periodontal diseases and hairy leukoplakia are typically seen in individuals with severe immune deficiency who do not receive ART [3].

Successful treatment with ART suppresses HIV replication resulting in significantly reduced mortality and morbidity and prevention of HIV transmission. The goal of successful ART is suppression of viral replication below a clinically defined threshold (usually plasma viral load <50 copies/mL (cp/mL)). However, in Europe and North America up to 6.2% [4] do not achieve this threshold with use of ART and have low level viremia (LLV) defined as multiple viral loads between 50–1000 cp/mL [5, 6]. There is an ongoing debate whether LLV is produced due to activation of already infected immune cells or results from viral replication at specific anatomical sites where ART penetration is insufficient [7, 8]. To suppress viremia and to prevent development of antiviral resistance a switch to a high-genetic barrier antiviral compound, like boosted darunavir (DRV), is often considered in patients with LLV.

For HIV, the lower gastrointestinal tract is a well-studied viral reservoir [8]. The oral cavity as the most proximal part of the gastrointestinal tract, has been proposed as a viral reservoir as well [9]. Within the oral cavity, gingival tissue and lymphoid tissue can harbor a significant amount of HIV even in individuals receiving ART [10]. It is assumed that the presence of HIV in saliva, reflects either the presence of HIV infected cells in the salivary glands, tonsils or the periodontium [11]. In individuals with an HIV plasma viral load ≥2000cp/mL up to 64% has detectable HIV-RNA in saliva [12]. For individuals with low or suppressed plasma viremia it is not well known if HIV-RNA is also detectable in saliva. A measurable sign of oral compartmentalization would be higher levels of HIV in saliva than in plasma. This has been observed in a small number of subjects that had HIV-associated periodontal disease, gingival inflammation and were not receiving triple-drug ART [13].

Additionally, the oral cavity has its own unique set of physiological properties and barriers [14]. The oral mucosa forms a first-line protection against potential pathogens and consist of two types of barriers; a physiological barrier and a microbiological barrier [14]. The physiological barrier consists of tight junctions between cells preventing the flow of certain biological excretions such as saliva, water, ions, and other small molecules, including immunoglobulins and antimicrobial peptides through the barrier, creating different environments on either side of the barrier. Comparable with the lower gastro-intestinal tract, there is a microbiological barrier that consists of more than 700 bacterial species that live in a dynamic balance with the host and its oral immune system [15, 16].

When periodontitis arises, both the physical barrier and the microbiological-immune barrier can become disturbed, with an increase of a small subset of bacteria being strongly associated with periodontal disease. Examples include, but are not limited to, *Aggregatibacter actinomycetemcomitans (Aa)*, *Treponema denticola (Td)*, *Tannerella forsythia (Tf)*, *Fusobacterium nucleatum (Fn)*, *Porphyromonas gingivalis (Pg)*, *Prevotella intermedia (Pi)*, *Parviromonas micra (Pm)*. Some of these periodontopathogens can colonize teeth, form a biofilm (dental plaque) and affect gingival tissue, and in some instances manipulate mucosal host defenses or result in immune activation and inflammation [17]. Of relevance is that microbial translocation and inflammation have been shown to be elevated in individuals with periodontitis [18] and both microbial translocation and inflammation might activate HIV infected immune cells. In an *in vitro* study, it has been shown that the supernatant of *Pg* and *Fn* with short chain fatty acids can promote HIV transactivation of HIV in resting HIV infected macrophages, dendritic cells and T-cells [19] resulting in the production of new viral copies.

We hypothesize that periodontal inflammation and the presence of periodontal pathogens might result in to local and generalized immune activation triggering a cascade that can lead to activation of (latently) infected CD4^+^ cells, resulting in HIV production or replication and LLV in some patients.

## Methods

### Study design and individuals

To study the association between LLV and periodontitis we performed a substudy (this manuscript) as part of a study that investigated the dynamics of LLV in individuals who switched to a darunavir containing regimen at baseline and a primary endpoint at week 24 [20]. In total 11 patients participated in this substudy. The individuals were recruited from November 24, 2014, to December 13, 2017. Additionally, we compared findings with an age- and sex matched historical control group with suppressed HIV viremia (N = 11) [21].

Within the LLV group, measurements at baseline and week 24 (primary endpoint) were performed. At baseline individuals switched to a DRV containing ART regimen with the intention to further suppress viremia and prevent development of drug-resistance, without any periodontal intervention. LLV was defined as ≥2 viral loads between 50 and 1000 cp/mL in one year. Individuals with therapy nonadherence, major IAS-mutations for DRV (I47V; 150V; 154 M/L; L76V; 184V) [22], any signs of opportunistic infections or AIDS-defining illnesses or who were edentulous and individuals who had a history of radiation therapy in the head and neck region were excluded. The study was approved by the Institutional Review Board of the University Medical Center in Utrecht (14–018).

A historical control group was selected from a study with cross-sectional design that investigated periodontitis in individuals with suppressed HIV viremia based on age, sex, smoking, and diabetes. The measurements performed in this historical control group were similar. The historical control group used ART for ≥6 months, and had <50 copies/ml or undetectable virus in the last two measurements in plasma and was used to compare baseline findings in the LLV group. Recruitment was from August 28, 2015, to March 11, 2016. This study was approved by the Institutional Review Board of the University Medical Center in Groningen (2014/128) [23].

### Collection of blood and saliva samples

In the LLV group, samples were collected at baseline and week 24. Blood samples were collected in EDTA-tubes (20 mL) and Na-heparin-tubes (40 mL). Unstimulated whole saliva was collected by instructing individuals to abstain from eating and drinking one hour prior to sampling around noon. Before saliva collection individuals were instructed to rinse their mouth with water. At least 3 mL of whole saliva was collected by the spitting technique. By this technique accumulated saliva is periodically expectorated into a collecting tube [24, 25]. Collection time was at least five minutes or prolonged until a volume of at least 3mL was reached. Subsequently, 1.5 mL of saliva was transferred to a Universal Transport Medium (UTM) and the remainder of saliva (at least 1.5 mL) transferred to a protease inhibitor containing EDTA-tube. The protease inhibitor containing EDTA tubes were centrifuged at 1600 rpm and supernatant was stored at -80° C.

In the suppressed viremia group, blood samples were collected in EDTA-tubes and Na-heparin-tubes to compare virological and immunological data.

### Measuring periodontal inflammation

Our primary outcome was periodontal inflammation. In all individuals an extensive periodontal examination was performed by two experienced dental hygienists in which clinical periodontal inflammation was quantified. Measurements at baseline and week 24 were always performed by the same dental hygienist. This dental hygienist was not aware of the HIV-RNA when measuring the Periodontal Inflamed Surface Area (PISA). Clinical attachment loss (CAL), gingival recession and bleeding on probing (BOP) were measured and entered into a excel spreadsheet to calculate the PISA. The number of missing teeth was recorded. All teeth were examined on six sites per tooth with a periodontal probe (Williams probe 14 W, Hu-Friedy Mfg. Co., LLC, UK). The measurements were entered in a spreadsheet to calculate the periodontal inflamed surface area (PISA) [26]. Wisdom teeth were not included in the measurements. In the LLV group, a difference of more than 25% in PISA at 24 weeks compared to baseline was considered an increase or decrease. The presence of periodontitis was also defined according to the CDC-AAP case definition of periodontitis surveillance for epidemiologic studies. Mild periodontitis was recorded for cases with ≥2 interproximal sites with a CAL ≥3 mm and ≥2 interproximal sites with a Probing pocket depth (PPD) ≥4 mm (not on the same tooth) or 1 site with a PPD ≥ 5 mm. Individuals were classified as having moderate periodontitis in the presence of ≥2 interproximal sites with a CAL ≥4 mm (not on the same tooth) or ≥2 interproximal sites with a PPD ≥5 mm, also not on the same tooth. Severe periodontitis was recorded if the individuals had ≥2 interproximal sites with CAL ≥6 mm, not on the same tooth, and ≥1 interproximal site with a PPD ≥5 mm [27–29]. Measurement of periodontal inflammation was performed in a research setting and did not include an intervention or treatment advice. Patients were, however, afforded the liberty to seek dental care with their own dental care professional and continue their regular dental care visits.

### Periodontal pathogens

As secondary outcome we compared presence of periodontal pathogens. Within the LLV group, subgingival samples were taken from the deepest and/or bleeding pockets of each quadrant by using two sterile paper points per pocket. If bleeding in the periodontal pockets was absent, the mesial site of the first molar was chosen. In the absence of the first molar, the mesial site adjacent to the anterior tooth in the dental arch was selected instead. The paper-points were inserted in the pocket and left in place for 10 seconds. All eight paper points were pooled and sent to the Oral Microbiology Laboratory for molecular analyses (LabOral diagnostics, Houten, The Netherlands) to quantify the presence of *Aa*, *Td*, *Tf*, *Fn*, *Pg*, *Pi*, *Pm*. Bacterial plaque outcomes were categorized in five groups: category 0 (<10^3^ bacteria or colony forming units (CFU), category 1 (10^3^ and 10^4^ CFU), category 2 (10^5^ CFU), category 3 (10^6^CFU) and category 4 (≥10^7^CFU).

Within the suppressed viremia group, microbiological analysis of samples was performed by standard culturing protocol in the Oral Microbiology Laboratory of the UMCG [30, 31].

### HIV-RNA

As secondary outcome we compared HIV-RNA levels. Within LLV group, EDTA-tubes were processed within eight hours and plasma was stored in Eppendorf cups of 1.8mL at -80°C. HIV-RNA viral loads in plasma were measured by COBAS AmpliPrep/COBAS Taqman HIV-1 assay. Detection of HIV-RNA in saliva was first verified. In short, the use of sputolysin or Phosphate Buffered Saline (PBS) was verified with 8 saliva samples (1.5 mL) of HIV-negative volunteers in UTM that was spiked with Electron Microscopy counted HIV-HXB2 in a concentration of 10^4^ cp/mL. Spiked saliva samples of HIV negative donors were either diluted in PBS (1:1) or diluted 1:10 with sputolysin, with comparable levels of HIV-RNA for both methods. The lower limit of quantification (LLOQ) of HIV-RNA levels in saliva was 20 cp/mL. Levels between 50-1000cp/mL were defined as LLV. When HIV viremia was detected, but below the threshold of 50 cp/mL it was defined as residual viremia. All samples were tested simultaneously to reduce test variation. Two saliva samples were retested using sputolysin after failure to detect HIV-RNA due to clots in the samples. Within the suppressed viremia group EDTA-tubes were processed within eight hours and plasma was stored in Eppendorf cups of 1.8mL at -80°C. HIV-RNA viral loads in plasma were measured by Abbott RealTime HIV-1 Viral Load assay.

### Immune markers of activation and inflammation

As secondary outcome we compared immune markers. Within the LLV group, a multiplex Luminex assay was used to detect a variety of immune and inflammation markers: IL-6, IL-1b, IP-10, MCP-1 (CCL-2), MIP-1a (CCL-3), sICAM-1, sCD14 and sCD163 in saliva and plasma from baseline and week 24. Immune activation was evaluated at baseline and week 24 in plasma by cell-bound makers using Fluorescent Activated Cell Sorting (FACS). Briefly, cryopreserved PBMCs were thawed with RPMI 20% FCS and subsequently used for flow cytometric analysis. Cells were washed using PBS and incubated with monoclonal antibodies against CD4, CD8 and markers of immune activation CD38 and HLA-DR. Fluorescence minus one controls were used to define positive gates for the expression of different proteins. Analysis was performed with FlowJo Software. Differences of more than 25% between two measurements were considered relevant to include as an increase or decrease.

Within the suppressed viremia group, serum concentrations of the inflammation markers CRP, IL-6, CXCL-10, and the microbial translocation and inflammation markers sCD14, LPS, and sCD163, were assessed with ELISA. IL-6, LPS, CXCL-10 (R&D systems Minneapolis, MN), sCD14 and sCD163 (Themo Scientific, Waltham, MA), were measured according to the Dynex DS-2 system manufacturer’s protocol. To measure cellular markers, absolute numbers of CD3+, CD4+, and CD8+ T-cells and measured using the MultiTest TruCount method with MultiTest reagents directed at CD45/3/4/8 (Becton Dickinson).

Protocol differences between the LLV and suppressed viremia group were solved by only including parameters that were measured in both groups in the analyses.

### Statistical analysis

All data were analyzed using SPSS Statistics version 27.0. Data was not normally distributed; therefore, we used the Wilcoxon-signed rank test for analyzing differences within parameters on T = 0 and T = 24 and Mann-Whitney U to compare groups. Correlations were determined using the Spearman’s rank correlation coefficient. For all analyses, p<0.05 was defined as significant. Figures were made with Graphpad Prism (v.10.1.0). To allow a valid and balanced comparison, the suppressed viremia group was only compared with baseline data from the LLV group and not with week 24, because there was no longitudinal data from the control group

## Results

An overview of all 22 individuals is given with measurements at baseline (LLV and control) and week 24 (LLV only) in Table 1. In Table 2 differences between baseline and week 24 for the LLV group are presented on an individual level.

**Table 1:** Characteristics of 11 individuals with suppressed viremia at baseline and LLV at baseline and 24 weeks.

Characteristics		<50cp/mL group	LVV-group (50-1000cp/mL)	
Baseline or week 24		Baseline	Baseline	Week 24
N (%)		11	11	11
Male		10 (90.9%)	10 (90.9%)	9 (90.9%)
Individuals with known diabetes mellitus		2	0	0
Tobacco use	Current	4 (36.3%)	5 (45.5%)	6 (54.5%)
	Never	2 (18.1%)	3 (27.3%)	3 (27.3%)
	Former smokers	5 (45.5%)	3 (27.3%)	2 (18.2%)
Does the participant visit the dentist? *	No	0	1 (9.1%)	1 (9.1%)
	Yes, regularly	10 (90.9%)	9 (81.8%)	9 (81.8%)
	Only by complaints	1 (9.1%)	1 (9.1%)	-
Classification periodontitis	Moderate	3 (27.3%)	4 (40%)	5 (45.5%)
	Severe	8 (72.7%)	7 (63.5%)	6 (54.5%)
Present bacterial flora	*Aa*	0	0	0
	*Pg*	5 (45.5%)	3 (27.3%)	4 (36.4%)
	*Pi*	5 (45.5%)	3 (27.3%)	5 (45.5%)
	*Tf*	11 (100%)	10 (90.9%)	11 (100%)
	*Pm*	11 (100%)	10 (90.9%)	11 (100%)
	*Fn*	11 (100%)	11 (100%)	11 (100%)
Median PISA, mm^2^ (IQR)		1000.4 (640.6–1347.0)	1014.1 (293.2–1237.9)	818.9 (585.0–1077.1)
Mean PISA, mm^2^		1197.9 (928.7)	856.3 (496.2)	933.7 (581.0)
Detectable HIV-RNA in saliva (N)		-	2	3
Individuals with LLV at sampling		0	8	4
**Mean (SD)**				
**Mean HIV-RNA (cp/mL) (SD)**		3.64 (12.1)	152.6 (292.2)	27 (45.3)
**Median HIV-RNA (cp/mL) (IQR)**		0.0 (0.0–0.0)	53.0 (0.0–146.0)	00.0 (0.0–52.0)
Age in years		51.7 (6.4)	51.4 (6.6)	-
BMI (kg/m^2^)		24.5 (2.9)	25.0 (5.8)	-
CD4+T-cells (10^9^/L)		863.8 (410.7)	577.3 (300.0)	-
Activated CD4 cells (total) (%)		1.05 (0.9)	3.1 (1.4)	3.1(2.4)
Activated CD8 cells (total) (%)		1.37 (0.8)	2.6 (1.2)	2.7 (1.5)
Probing pocket depth mm		3.3 (0.9)	3.4 (0.7)	3.4 (0.9)
Recession		1.7 (0.6)	1.4(1.1)	1.7 (1.0)
Clinical attachment level mm		5.0 (1.2)	5.0 (1.6)	5.1 (1.7)
Median number of elements present** **(IQR)**		24 (21–28))	25 (22–27)	25 (22–27)
Saliva flow mL/min*		1.52 (0.7)	(N = 10) 1.04 (0.5)	(N = 8) 1.07 (0.3)
Patient reported importance of dental health (VAS)		8.8 (1.6)	8.9 (1.3)	9.1 (1.6)*

* = Data not available for all study participants

** = including wisdom teeth

**Table 2:** Overview of differences periodontal inflamed surface area and other virological, bacteriological and immunological parameters per individuals from LLV group at baseline and 24 weeks.

Subject ID		PISA (mm^2^)	HIV-RNA plasma (cp/mL)	HIV RNA saliva	Subject ID	Factors	Periodontal pathogens	Immunological parameters (blood)	Immunological parameters (saliva)
						increase↑			
				(cp/mL)		decrease ↓			
						equal ≈			
1	**BL**	522.47	1010	49	1	**↑**	Pg, Pm	IL-1β	none
(L-9)	**WK24**	585.04	53	20	(L-9)	**↓**	Tf, Fn, Td	IP-10; sCD163; CD4_tot_CD38^+^HLA-DR^+^; CD8_mem_CD38^+^HLA-DR^+^	none
	**Category**	Similar	pLLV	Decrease		**≈**	All others	All others	All others
2	**BL**	293.21	146	0	2	**↑**	Pi	IL-1β; sCD14; CD4_tot_CD38^+^HLA-DR^+^;	MCP-1; sCD14; sCD163
(L-23)					(L-23)				
								CD8_tot_CD38^+^HLA-DR^+^;	
								CD8_mem_CD38^+^HLA-DR^+^	
	**WK24**	309.70	0	0		**↓**	none	none	IL-1β; IP-10
	**Category**	Similar	Responder	No change		**≈**	All others	All others	All others
3	**BL**	244.81	0	0	3	**↑**	none	IP-10	IL-1β; MIP-1α; IP-10; sICAM; sCD14
(L-1)					(L-1)				
	**WK24**	818.91	0	0		**↓**	Fn	none	sCD163
	**Category**	Increase	Responder	No change		**≈**	All other	All other	All other
4	**BL**	1014.13	219	0	4	**↑**	Pi, Tf, Pm, Fn	sCD163; sICAM; sCD14; CD4_tot_CD38^+^HLA-DR^+^;	IL-1β; sCD14
(L-2)					(L-2)				
								CD4_mem_CD38^+^HLA-DR^+^;	
								CD8_tot_CD38^+^HLA-DR^+^	
	**WK24**	1007.14	144	45		**↓**	none	none	IL-6; MCP-1; IP-10; sCD163
	**Category**	Similar	pLLV	Increase		**≈**	All others	All others	All others
5	**BL**	1237.91	0	0	5	**↑**	None	none	none
(L-16)	**WK24**	1077.13	48	0	(L-16)	**↓**	Pg, Tf, Fn	IL-6; IP-10; sCD163; sICAM;	IL-1β; IL-6; MCP-1; MIP-1α; IP10; sCD163; sICAM; sCD14
								CD4_tot_CD38^+^HLA-DR^+^;	
								CD4_mem_CD38^+^HLA-DR^+^;	
								CD8_tot_CD38^+^HLA-DR^+^;	
								CD8_mem_CD38^+^HLA-DR^+^	
	**Category**	Similar	Responder	No change		**≈**	All others	All others	All others
6	**BL**	1054.95	41	0	6	**↑**	Fn	IL-1β; MCP-1, MIP-1α, IP-10; sCD163; sICAM	IL-1β; sCD14
(L-30)					(L-30)				
	**WK24**	756.51	0	0		**↓**	Pm	sCD14;	sCD163
								CD4_tot_CD38^+^HLA-DR^+^;	
								CD4_mem_CD38^+^HLA-DR^+^;	
								CD8_tot_CD38^+^HLA-DR^+^;	
								CD8_mem_CD38^+^HLA-DR^+^	
	**Category**	Decrease	Responder	No change		**≈**	All others	All others	All others
7	**BL**	1715.13	76	0	7	**↑**	Tf, Pm, Fa	None	IL-6; MIP-1α; IP-10
(L-10)	**WK24**	2342.74	0	0	(L-10)	**↓**	None	CD4_tot_CD38^+^HLA-DR^+^;	IL-1b; MCP-1; sICAM
								CD4_mem_CD38^+^HLA-DR^+^;	
								CD8_tot_CD38^+^HLA-DR^+^;	
								CD8_mem_CD38^+^HLA-DR^+^	
	**Category**	Increase	Responder	No change		**≈**	All others	All others	All others
8	**BL**	166.61	40	0	8	**↑**	None	CD4_mem_CD38^+^HLA-DR^+^;	MCP-1; MIP-1α; IP-10; sCD163; sCD14
(L-15)					(L-15)				
	**WK24**	222.04	0	20		**↓**	Tf	IL-1β; IL-6; sCD163	IL-6
	**Category**	Increase	Responder	Increase		**≈**	All others	All others	All others
9	**BL**	1312.21	0	0	9	**↑**	Pm; Fn	None	None
(L-17)	**WK24**	1453.59	0	0	(L-17)	**↓**	None	IL-1β; sICAM; sCD14	IL-1β; IL-6; MCP-1; MIP-1α; IP-10; sCD163; sICAM; sCD14
	**Category**	Similar	Responder	No change		**≈**	All others	All others	All others
10	**BL**	1024.71	93	22	10	**↑**	Pi; Tf	IL-6: IP-10; sCD14;	IL-1β; IL-6; sCD163; sCD14; sICAM; IP-10
(L-11)					(L-11)			CD8_mem_CD38^+^HLA-DR^+^	
	**WK24**	942.51	52	0		**↓**	Fn	CD4_tot_CD38^+^HLA-DR^+^;	None
								CD4_mem_CD38^+^HLA-DR^+^	
	**Category**	Similar	pLLV	Decrease		**≈**	All others	All others	All others
11	**BL**	833.16	53	0	11	**↑**	None	IL-1β; MCP-1; CD8_tot_CD38^+^HLA-DR^+^;	sCD163; IL1β; IL-6
(L-29)					(L-29)				
	**WK24**	755.50	0	0		**↓**	None	IP-10	MCP-1; IP-10; sCD14
	**Category**	Similar	Responder	No change		**≈**	All	All others	All others

Difference between baseline and week 24 on an individual level from LLV group. BL = baseline; W24 = week 24; ↑ = increase (>25% change); ↓ = decrease (>25%change); ≈ = similar (<25% change). pLLV = persistent Low Level Viremia. *Pg = Porphyromonas gingivalis; Pm = Parviromonas micra; Fn = Fusobacterium nucleatum; Tf = Tannerella forsythia; Fa = Filifactor alocis; Pi = Prevotella intermedia; Td = Treponema denticola*.

### Periodontal inflammation

**Fig 1:**
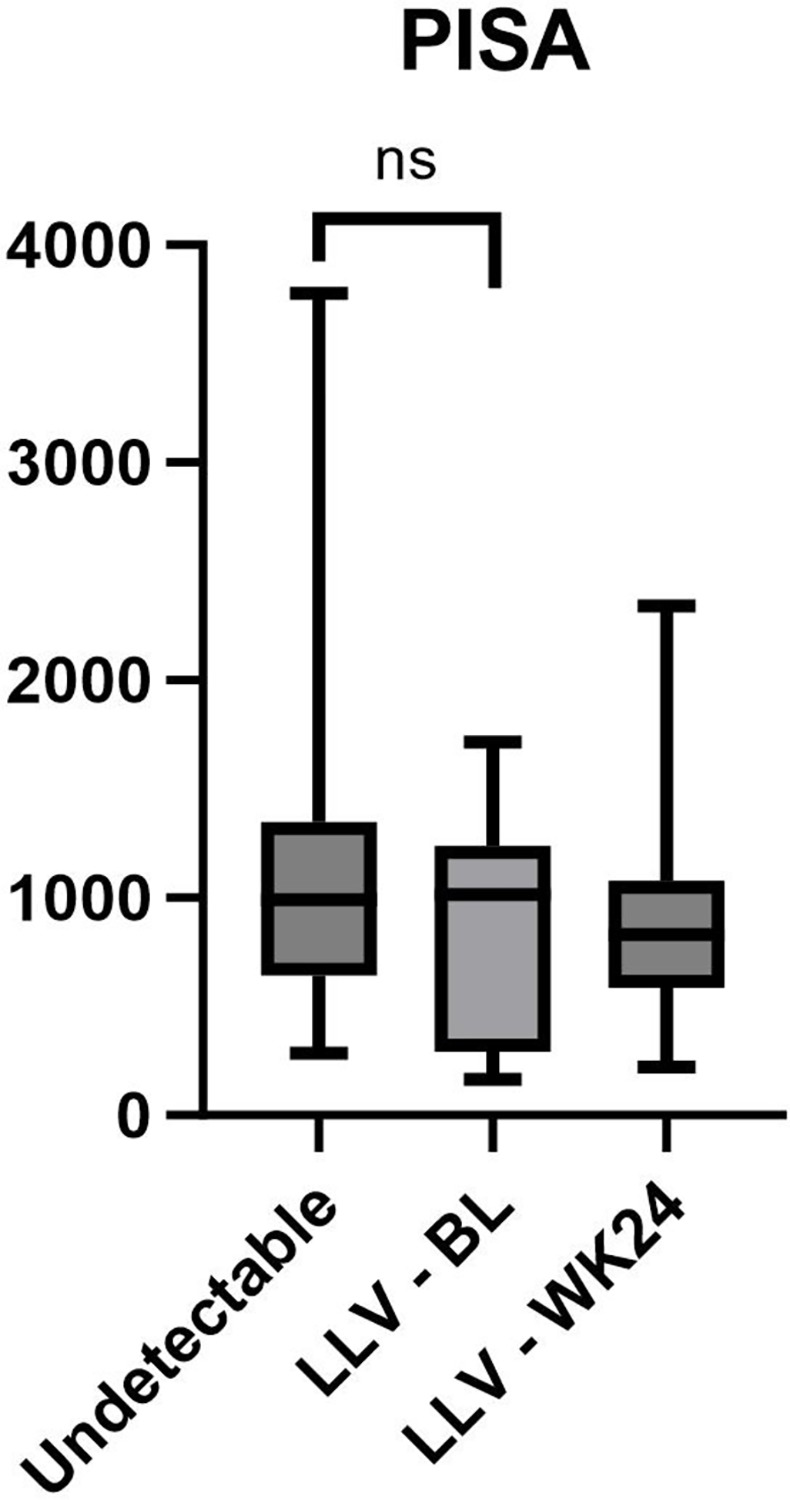
Comparison of median PISA in group with undetectable viral load and LLV group. PISA between these groups is not significantly (ns) different (p = 0.48).

All individuals in both the LLV-group and control group had moderate (Suppressed N = 3; LLV-BL N = 4; LLV-WK24 N = 5) to severe periodontitis (Suppressed N = 8; LLV-BL N = 7; LLV-WK24 N = 6) according to the CDC-AAP case definition of periodontitis surveillance for epidemiologic studies (see Table 1). When the control group and LLV-group were compared no significant difference was seen at baseline (p = 0.48, Fig 1).

Within the LLV group three individuals (subject 3, 7 and 8) had deterioration of PISA (see Table 2). In one individual (subject 6) PISA improved and in 7 individuals (subject 1, 2, 4, 5, 9, 10, 11) PISA remained similar at baseline and week 24 (see Table 2). Within the LLV group, median PISA did not change significantly (p = 0.72) with median 1014 mm^2^ at baseline and 818.9 mm^2^ at week 24.

### HIV-RNA

To understand the dynamics of HIV-RNA and the role of the oral cavity in the LLV-group, we compared plasma and saliva viral loads after switch of ART.

At screening, all individuals within the LLV group had LLV in plasma, but at baseline 5 individuals (subject 3, 5, 6, 8, 9) had a viral load <50 cp/mL without any intervention, demonstrating that LLV can have variation around the clinically used cut-off of 50 cp/mL. Of the 11 individuals three had persistent LLV (subject 1, 4, 10) and eight had a viral load <50cp/mL at week 24 (subject 2, 3, 5, 6, 7, 8, 9, 11) in plasma. In four individuals very low levels of HIV-RNA (<50cp/mL) were detected in saliva at baseline (subject 1 and 10) or at week 24 (subject 1, 4, 8). Of interest is that HIV-RNA in saliva is mostly seen in individuals with persistent plasma LLV (subject 1, 4, 10) and in only one person who became virologically suppressed (subject 8). Within the LLV group, viral load in plasma did not change significantly (p = 0.168) with a mean of 153 cp/mL at baseline and 27 cp/mL at week 24. Mean viral load in saliva did not change significantly (p = 0.580) with 6.5 cp/mL at baseline and 7.8 cp/mL at week 24. HIV-RNA in saliva and plasma was not correlated (p = 0.83) despite detecting HIV-RNA in saliva mostly in individuals with persistent LLV.

All individuals in the control group had a viral load <50cp/mL in blood. Saliva viral load was not measured in the control group.

### Periodontal pathogens

To understand the association of periodontal pathogens and periodontitis we compared the presence of several periodontal pathogens in both the LLV and control group. Periodontal pathogens were observed in both the LLV and control group in all individuals. Within the LLV group *Fn* was seen in all individuals at baseline and week 24 (Table 1). *Tf* and *Pm* were frequently observed with 91% at baseline and 100% at week 24. *Pg* and *Pi* were seen in 3 individuals at baseline and at week 24 in 4 individuals (*Pg*) or 5 individuals (*Pi*). In contrast *Aa*, was not observed at all. Changes in bacterial plaques on an individual level for the LLV group can be seen in Table 2. Of interest is that an increase in PISA is not always associated with an increase in periodontal pathogens (subject 3 and 8). In subject 7, who had a higher PISA at week 24 there was an increase of *Tf*, *Pm*, *Fa*. However, an increase of these bacteria is not in all cases accompanied with an increase in PISA (subject 1, 4, 9, 10).

Within the control group *Fn*, *Tf*, *Pm* were seen in all individuals and *Pg* and *Pi* in 5 individuals.

### Immunological parameters

**Fig 2:**
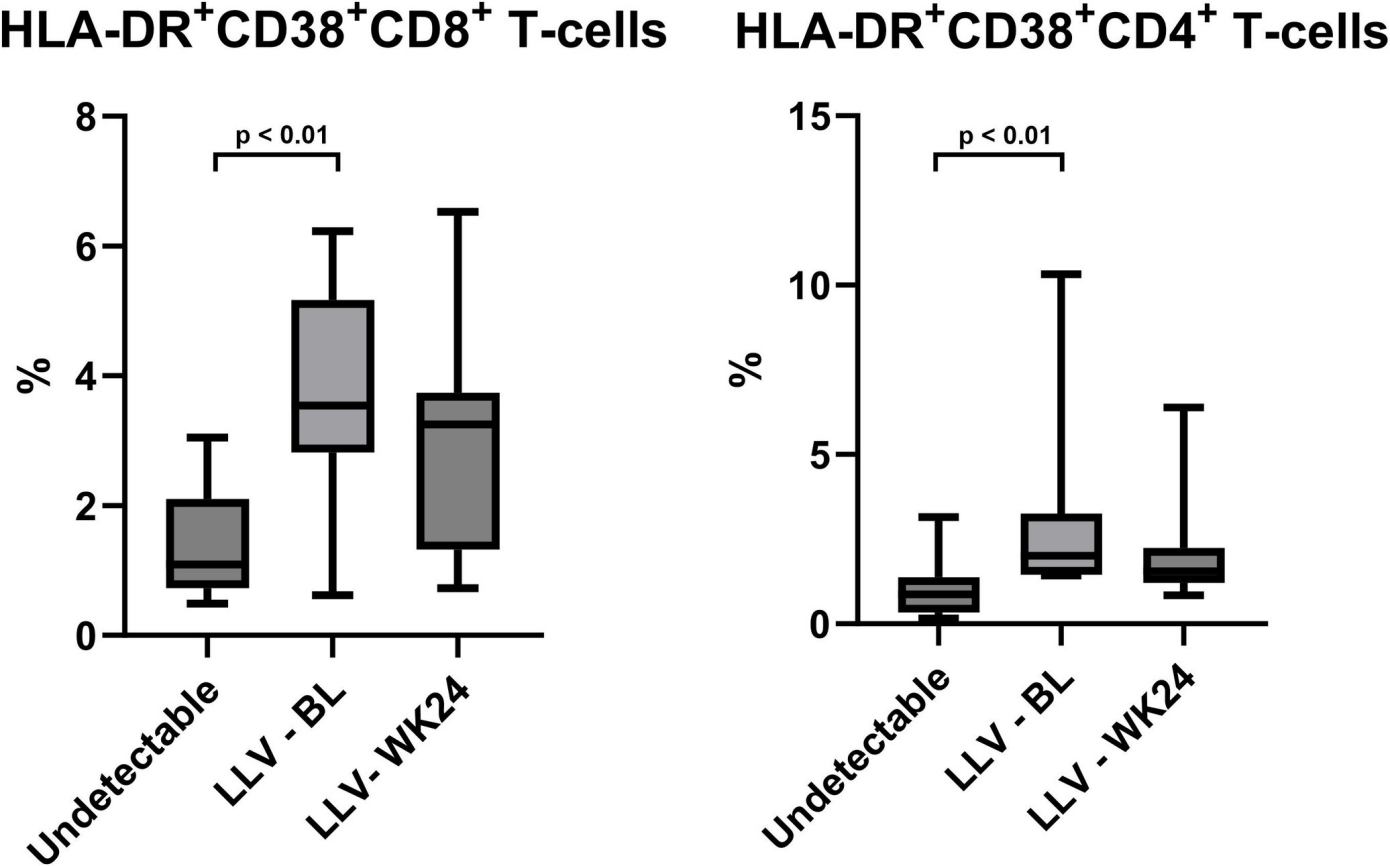
Activated HLA-DR^+^CD38^+^ CD8^+^ T cells (left) and CD4^+^ T cells (right). A significant difference between activated cells is seen with undetectable viral load and LLV group, with higher activation levels in LLV group. **p<0.01; ***p<0.01.

To understand differences in immune activation as a result of LLV, we compared immune markers from the LLV group at baseline and control group. Total activated CD4^+^ cells and CD8^+^ cells were significantly higher in the LLV group than in the suppressed viremia group (p = <0.01) (Fig 2).

To investigate changes in immune activation and inflammation after switch of ART, we compared baseline and week 24 in the LLV group. Three individuals (subject 3, 7 and 8) with an increase in PISA during follow-up all had an increase in MIP1a and IP-10 in saliva. However, IP-10 was found to be increased also in individuals without a change in PISA (individual 9 or 10) and the same is true for MIP1a (subject 9). The individuals with a decrease in PISA had lower levels of sCD163 at week 24 in saliva. However, a decrease in sCD163 was also seen in individuals with a deteriorating PISA (subject 3) or when PISA remained similar (subject 2, 4 and 9). Although PISA improved in subject 6, several immune markers in blood (IL-1b; MCP-1; MIP-1a; IP-10; sCD163) became higher. We could not identify particular markers of interest in individuals with persistent LLV nor for the individuals with HIV-RNA in saliva. See Table 2.

We did not observe any differences between immunological parameters in saliva between baseline and week 24 within the LLV group. In plasma, we saw that median MCP-1 levels were higher at week 24 compared to baseline (BL = 122 pg/mL; week 24 = median 140 pg/mL (p = 0.033). This was however not significant when a Bonferroni correction for multiple testing was performed. In saliva higher levels were seen for IL-1β and MCP-1, whereas in plasma higher levels of MIP-1α, sCD163, sICAM, sCD14 are seen (see S1 File).

## Discussion

This multicenter prospective study compared periodontal inflammation, virological and immunological parameters in plasma and saliva in individuals with HIV low level viremia at baseline and week 24. A historical control group with suppressed viremia was used to compare periodontal inflammation, virological and immunological parameters in plasma at one time point. We hypothesized that the periodontium might be involved in LLV, either directly or indirectly.

First of all, we confirm that HIV-RNA can be detected in saliva. In our study we found that in 4 out of 11 individuals HIV-RNA could be detected in saliva, but at residual levels (RNA detected, but <50cp/mL). Others have also shown that HIV-RNA [10, 13] and DNA [32] can be detected in saliva or gingival crevicular fluid. While HIV can be detected in saliva it is not considered a relevant route of HIV transmission [33]. Levels of HIV in saliva are generally low as a result of ART, salivary antiviral factors and Immunoglobins that neutralize HIV [34]. Oral HIV hypersecretion is a rare phenomenon but has been described in individuals with periodontal disease and not receiving ART [13]. In our study, oral hypersecretion in saliva was found in none of the individuals, but it is of interest that 3 out of 4 individuals in which HIV-RNA was detected in saliva were individuals with persistent LLV. Unfortunately, we could not investigate the presence of HIV-RNA in saliva from the suppressed viremia group because the saliva was not available for this analysis.

Suggestive for oral compartmentalization are comparative studies that have demonstrated detection of p24 antigen in gingival biopsies [10] and a 100 to 10000-fold higher RNA levels in tonsil biopsies compared with plasma [35]. In our study we did not perform tissue biopsies because it was considered too invasive and because there is a high risk of sample contamination with blood when biopsies are taken.

In previous literature, it has been described that activated HLA-DR^+^CD38^+^ CD8^+^ and CD4^+^ T cells as markers of immune activation are higher in the group of untreated individuals with HIV viremia, compared to HIV uninfected and suppressed control group [36, 37]. In line with previous studies, we also found that that activated HLA-DR+CD38+ CD8+ and CD4+ T cells are higher in the LLV group compared to suppressed viremia group. Also, in agreement with previous literature is that periodontal inflammation is a common problem in PWH [23]. As known, periodontitis is characterized by the recruitment of neutrophils and lymphocytes in gingival tissue and results in clinical signs such as gingival bleeding, attachment loss and periodontal pocket formation [38]. Additionally, inflammatory markers such as IL-1b, IL-6 and TNF-α are associated with periodontitis [39, 40] and HIV [41–43], and possibly elicit HIV reactivation [44]. However, in our study we could not confirm that these or other immune markers in saliva or serum were associated with periodontitis or HIV in either the LLV or control group.

Periodontitis can be considered a dysbiosis, with enhanced virulence of certain pathogens in the context of a polymicrobial infection [45]. The role of certain periodontal pathogens is of particular interest as some bacteria or bacterial products can activate the HIV promoter in mature dendritic cells [46] or macrophages [47] and T cells [48] *in vitro*. Short-chain fatty acids, like butyric acid, from gram-negative bacteria such as *Pg* and *Fn* can strongly induce transactivation in latently infected T cells in a dose dependent manner *in vitro* [19]. Whether this also leads to reactivation *in vivo* is not known, but in one study p24 antigen was detected in gingival tissue in individuals with periodontitis and undetectable HIV RNA in plasma [10]. Although we did not perform biopsies, we did observe presence of HIV-RNA in saliva in 4 individuals, but we could not relate this to the presence of certain bacterial species nor PISA. Although a positive correlation between periodontal disease and HIV viral load in plasma has been described [49], this was not seen in our study, possibly because of the lower range of HIV-RNA in our study.

The major limitation of this study is the small sample size which impacts statistical power, which limits generalizability and the possibility to properly analyze confounding factors (e.g. smoking, diabetes). Another limitation is that two similar, yet not identical research protocols and methodology were used for the LLV- and control group, in particular no saliva HIV-RNA measurements in the control group. Nevertheless, we provided a valuable and unique overview of individuals with HIV LLV and suppressed viremia and its relation with immune activation markers, periodontal inflammation and periodontal pathogens and can be of value for future research. Future research should be performed in a larger sample size and use robust statistical methods to investigate the impact of periodontal inflammation on HIV LLV.

In our study we did not find evidence to support the involvement of the periodontal inflammation in the pathogenesis of LLV. Although the absence of evidence is not equal to evidence of absence, a strong association between periodontal inflammation and LLV is unlikely, because periodontitis is a very common problem in PWH on ART and LLV is much less frequently observed. HIV-RNA could be detected at very low levels in saliva in some individuals participating in the study, but there was no evidence for HIV-RNA hypersecretion or HIV reactivation due to periodontal inflammation or periodontal pathogens. A decreasing level of already low levels of HIV-RNA in saliva or plasma was not accompanied by a lower PISA. As we did not find an association between PISA, immune activation markers, periodontal pathogens and HIV-RNA levels, we would like to conclude that periodontal inflammation is an unlikely source of HIV LLV in plasma in this population.

## Supporting information

S1 File Soluble immunological markers in saliva and plasma. *N = 10; L-9 saliva in protease inhibitor containing EDTA-tube not available.
(DOCX)
