# Supplementary material for: Periodontal inflammation as a potential driver of HIV low level viremia
Source: PLoS One. 2024 Jun 17;19(6):e0305641. doi: 10.1371/journal.pone.0305641 (PMC11182545; doi:10.1371/journal.pone.0305641)
Supplement: S1 File — *N = 10; L-9 saliva in protease inhibitor containing EDTA-tube not available. (DOCX) [file pone.0305641.s001.docx]

**Supplement 1. Soluble immunological markers in saliva and plasma**

| **Plasma** | **Baseline**  **Median (IQR)** | **Week 24**  **Median (IQR)** | **p-value** |
| --- | --- | --- | --- |
| IL-1β (pg/mL) | 2.1 (0.7-2.4) | 2.1 (1.8-2.6) | 0.656 |
| IL-6 (pg/mL) | 10.8 (0.0-13.2) | 11.7( 9.0-15.0) | 0.333 |
| MCP-1 (pg/mL) | 121.7 (79.5-160.5) | 139.7 (100.1 -166.6) | 0.033 |
| MIP-1α (pg/mL) | 38.3 (36.3-43.3) | 40.1 (37.0-43.8) | 0.441 |
| IP-10 (pg/mL) | 314.4 (219.9-400.1) | 276.2 (178.4- 411.6) | 0.424 |
| sCD14 (pg/μL) | 1790.0 (1430.4-2070.0) | 1911.7 (1326.9 – 2420.0) | 1.000 |
| sCD163 (pg/mL) | 4389.5 (2974.8-6566.7) | 3802.5 (3145.4- 4986.2) | 0.929 |
| sICAM (pg/μL) | 274.0 (207.9 - 327.1) | 242.6 (192.5-325.8) | 0.790 |

| **Saliva** | **Baseline***  **Median (IQR)** | **Week 24**  **Median (IQR)** | **p-value** |
| --- | --- | --- | --- |
| IL-1β (pg/mL) | 70.7 (17.9-130.1) | 52.2 (26.3-242.0) | 0.799 |
| IL-6 (pg/mL) | 14.9 (8.0- 46.0) | 13.4 (3.1-30.8) | 0.779 |
| MCP-1 (pg/mL) | 320.1 (223.1-1288.9) | 249.5 (178.9-1033.0) | 0.260 |
| MIP-1α (pg/mL) | 0.0 (0.0-24.2) | 12.1 (0.0-31.1) | 0.917 |
| IP-10 (pg/mL) | 288.3 (49.9-1075.9) | 545.6 (3.6.711.8) | 0.445 |
| sCD14 (pg/μL) | 54.1 (5.4-117.2) | 68.6 (13.8 – 88.0) | 0.445 |
| sCD163 (pg/mL) | 491.8 (161.4-1291.0) | 727.2 ( 442.9-1544.7) | 0.575 |
| sICAM (pg/μL) | 3.0 (1.4 - 7.4) | 4.4 (1.3-6.6) | 0.646 |

| **Baseline** | **Saliva***  **Median (IQR)** | **Plasma**  **Median (IQR)** | **p-value** |
| --- | --- | --- | --- |
| IL-1β (pg/mL) | 70.7 (17.9-130.1) | 2.1 (0.7-2.4) | 0.017 |
| IL-6 (pg/mL) | 14.9 (8.0- 46.0) | 10.8 (0.0-13.2) | 0.069 |
| MCP-1 (pg/mL) | 320.1 (223.1-1288.9) | 121.7 (79.5-160.5) | 0.009 |
| MIP-1α (pg/mL) | 0.0 (0.0-24.2) | 38.3 (36.3-43.3) | 0.017 |
| IP-10 (pg/mL) | 288.3 (49.9-1075.9) | 314.4 (219.9-400.1) | 0.799 |
| sCD14 (pg/μL) | 54.1 (5.4-117.2) | 1790.0 (1430.4-2070.0) | 0.005 |
| sCD163 (pg/mL) | 491.8 (161.4-1291.0) | 4389.5 (2974.8-6566.7) | 0.005 |
| sICAM (pg/μL) | 3.0 (1.4 - 7.4) | 274.0 (207.9 - 327.1) | 0.005 |

| **Week 24** | **Saliva**  **Median (IQR)** | **Plasma**  **Median (IQR)** | **p-value** |
| --- | --- | --- | --- |
| IL-1β (pg/mL) | 52.2 (26.3-242.0) | 2.1 (1.8-2.6) | 0.004 |
| IL-6 (pg/mL) | 13.4 (3.1-30.8) | 11.7( 9.0-15.0) | 0.386 |
| MCP-1 (pg/mL) | 249.5 (178.9-1033.0) | 139.7 (100.1 -166.6) | 0.006 |
| MIP-1α (pg/mL) | 12.1 (0.0-31.1) | 40.1 (37.0-43.8) | 0.008 |
| IP-10 (pg/mL) | 545.6 (3.6.711.8) | 276.2 (178.4- 411.6) | 0.859 |
| sCD14 (pg/μL) | 68.6 (13.8 – 88.0) | 1911.7 (1326.9 – 2420.0) | 0.003 |
| sCD163 (pg/mL) | 727.2 ( 442.9-1544.7) | 3802.5 (3145.4- 4986.2) | 0.003 |
| sICAM (pg/μL) | 4.4 (1.3-6.6) | 242.6 (192.5-325.8) | 0.003 |

*N=10; L-9 saliva in protease inhibitor containing EDTA-tube not available
